# New Insights into the Regulation of mTOR Signaling via Ca^2+^-Binding Proteins

**DOI:** 10.3390/ijms24043923

**Published:** 2023-02-15

**Authors:** Yuna Amemiya, Masatoshi Maki, Hideki Shibata, Terunao Takahara

**Affiliations:** Department of Applied Biosciences, Graduate School of Bioagricultural Sciences, Nagoya University, Nagoya 464-8601, Japan

**Keywords:** mTOR, Ca^2+^ mobilization, calmodulin, TSC2

## Abstract

Environmental factors are important regulators of cell growth and proliferation. Mechanistic target of rapamycin (mTOR) is a central kinase that maintains cellular homeostasis in response to a variety of extracellular and intracellular inputs. Dysregulation of mTOR signaling is associated with many diseases, including diabetes and cancer. Calcium ion (Ca^2+^) is important as a second messenger in various biological processes, and its intracellular concentration is tightly regulated. Although the involvement of Ca^2+^ mobilization in mTOR signaling has been reported, the detailed molecular mechanisms by which mTOR signaling is regulated are not fully understood. The link between Ca^2+^ homeostasis and mTOR activation in pathological hypertrophy has heightened the importance in understanding Ca^2+^-regulated mTOR signaling as a key mechanism of mTOR regulation. In this review, we introduce recent findings on the molecular mechanisms of regulation of mTOR signaling by Ca^2+^-binding proteins, particularly calmodulin (CaM).

## 1. Introduction

It is essential for eukaryotes to sense environmental changes, such as changes in nutrients, growth factors, hormones, and stressors. This permits the organism to modulate the balance between anabolism and catabolism to regulate proper cell growth and proliferation. A key mediator in these roles is a large protein kinase called mechanistic target of rapamycin (mTOR). mTOR is a mammalian ortholog of TOR that was first identified in yeast in 1991 [1]. As suggested by its name, mTOR is a functional cellular target of the immunosuppressant and anticancer drug rapamycin [1,2,3].

**Figure 1:**
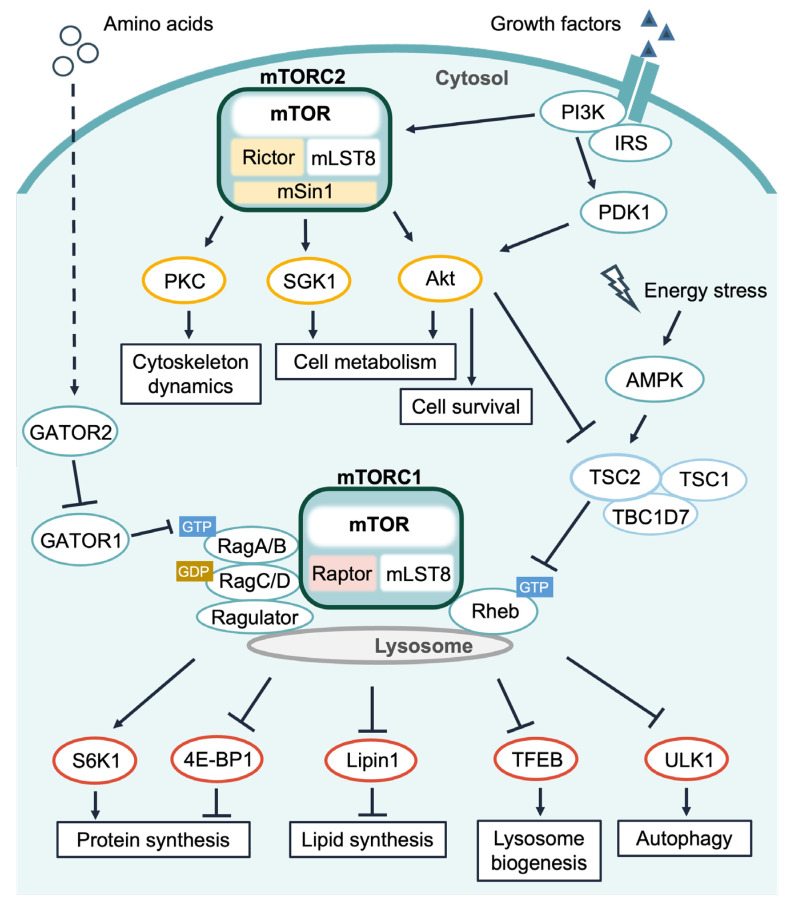
General functions of mTOR signaling. mTORC1 is primarily composed of mTOR, Raptor, and mLST8. mTORC2 is composed of mTOR, Rictor, mSin1, and mLST8. Multiple stimuli, including amino acids, growth factors, and energy stress regulate mTORC1 activity via the GATOR2–GATOR1–Rag GTPases axis and the TSC2–Rheb GTPase axis. mTORC1 mainly functions to activate anabolism, protein synthesis, or lipid synthesis and to inhibit catabolism, lysosome biogenesis, or autophagy by directly phosphorylating various substrates (closed red curves in oval shapes). In contrast, mTORC2 is thought to respond to growth factors or insulin. Activated mTORC2 regulates cell survival, metabolism, and cytoskeleton dynamics by directly phosphorylating the AGC kinase protein family of Akt, PKC, and SGK1. Major direct substrates of mTORC2 are shown by closed orange curves in oval shapes.

mTOR forms two complexes: mTOR complex 1 (mTORC1) and mTOR complex 2 (mTORC2). These two complexes are functionally and structurally different and have important roles in distinct intracellular signaling pathways (Figure 1). mTORC1 contains three core components: mTOR, the regulatory-associated protein of mTOR (Raptor), and mammalian lethal with SEC13 protein 8 (mLST8). mTORC1 promotes protein synthesis and lipogenesis, while mTORC1 inhibits lysosome biogenesis and autophagy by phosphorylating its substrates [2,3,4,5,6,7]. In brief, mTORC1 regulates protein synthesis through directly phosphorylating Thr389 on p70 S6 kinase 1 (S6K1) and several sites on eukaryotic initiation factor 4E (eIF4E)-binding protein 1 (4E-BP1) [3,8]. S6K1 regulates translation initiation and elongation by activating factors that control translation initiation, such as eukaryotic initiation factor 4A (eIF4A) and initiation factor 4B (eIF4B) [9,10], or by inhibiting eukaryotic elongation factor 2 kinase (eEF2K), which interferes with polypeptide chain elongation [11,12]. In addition, eIF4E promotes the translocation of ribosomal subunits to mRNA by recognizing and binding to the 5′ cap structure of mRNA, and 4E-BP1 binds to and inhibits eIF4E. The phosphorylation of 4E-BP1 by mTORC1 cancels its inhibitory activity toward eIF4E by preventing the binding between 4E-BP1 and eIF4E, thereby promoting translation initiation. In addition, mTORC1 enhances the activation of sterol regulatory element-binding protein 1 (SREBP1), a key transcriptional factor of lipogenic genes by phosphorylating Lipin1, and modulates its nuclear localization [5]. In addition to promoting such anabolic processes, mTORC1 also regulates cell growth by inhibiting catabolism. For example, mTORC1 phosphorylates transcription factor EB (TFEB) and prevents its nuclear translocation, thereby inhibiting lysosome biosynthesis [6,13]. mTORC1 also phosphorylates unc-51-like autophagy-activating kinase 1 (ULK1) and autophagy-related protein 13 (Atg13), which are involved in the early stages of autophagy, thereby inhibiting autophagosome formation [7,14]. mTORC2 consists of four core subunits: mTOR, the rapamycin-insensitive companion of mTOR (Rictor), mLST8, and mammalian stress-activated protein kinase-interacting protein 1 (mSin1) [15,16]. Although a wide variety of mTORC1 substrates have been identified, information on mTORC2 substrates is still limited. mTORC2 is known to mainly phosphorylate cAMP-dependent, cGMP-dependent, and protein kinase C-type (AGC) kinases, including Akt (also known as protein kinase B, PKB), protein kinase C (PKC) family members, and serum- and glucocorticoid-induced kinases 1 (SGK1) [8]. mTORC2 activity regulates cell survival, metabolism, and cytoskeleton [3,17,18] (Figure 1).

mTORC1 activity is controlled by amino acids, growth factors, glucose, energy status, and stress [2,3,19]. The most characterized mechanism of mTORC1 activation by amino acids and growth factors involves two distinct small GTPases: Ras-related GTP-binding protein (Rag) GTPases and Ras homolog enriched in brain (Rheb) GTPase. Rag GTPases exist as heterodimers of RagA (or RagB) and RagC (or RagD) and localize to the lysosomal surface by interacting with a lysosome-anchored pentameric complex called Ragulator [20,21]. Amino acids promote the active forms of the Rag heterodimer, GTP-loaded RagA/B, and GDP-loaded RagC/D. The Rag heterodimer uses a unique intersubunit communication to rapidly respond to amino acid availability [22]. The binding of a first GTP molecule to one subunit of the dimer induces a conformational change that suppresses the association of a second GTP molecule to the other subunit. The active form of Rag GTPases can bind to Raptor, which promotes the lysosomal translocation of mTORC1 [2,3,19]. A recent study suggested different roles for Rag paralogues in the amino acid-mediated regulation of mTORC1 [23]. Luminal amino acids in lysosomes activate Rag GTPases via the association of vacuolar-type H^+^-ATPase (v-ATPase) and solute carrier family 38 member 9 (SLC38A9) with Ragulator [2,3,19,24,25]. The recruitment of mTORC1 at the lysosomal surface is believed to facilitate interaction with the mTORC1 activator Rheb GTPase [26,27] that is activated by growth factor signaling and amino acids. In addition to the well-characterized mechanism of mTORC1 activation, several reports have demonstrated that mTORC1 is activated in Golgi compartments, although the detailed mechanisms remain unclear [28,29,30]. Other detailed mechanisms of mTORC1 activation have been described in recent reviews [2,3,18,19]. Compared to mTORC1, the activators and mechanisms of mTORC2 are poorly understood, although it is thought that mTORC2 is mainly regulated by growth factors, including insulin (Figure 1).

Consistent with the diverse roles of mTOR signaling in controlling cellular metabolism and proliferation, novel regulatory mechanisms have recently emerged. Interestingly, mTOR signaling, especially mTORC1, is sensitive to changes in the intracellular concentration of calcium ions (Ca^2+^). It has been known for more than two decades that Ca^2+^ mobilization influences mTORC1 activity [31,32]. However, the detailed molecular mechanisms leading to mTORC1 have been unclear for a long time. Recently, many studies have suggested several molecular mechanisms of mTORC1 regulation by intracellular Ca^2+^ mobilization [19,33,34,35,36]. In addition, recent studies have suggested that Ca^2+^ signaling and subsequent regulation of mTORC1 signaling are the underlying basis for physiological and pathological cardiac hypertrophy [37,38].

In both cellular and physiological aspects as described above, understanding the molecular mechanism of Ca^2+^-mediated mTOR signaling is of great interest, because this signaling has fundamental roles in regulating cellular and physiological statuses. In this review, we summarize recent reports of Ca^2+^-mediated regulation of mTORC1 and mTORC2 signaling, with an emphasis on the Ca^2+^-binding protein calmodulin (CaM), the most-described Ca^2+^ sensor involved in mTOR signaling.

### 1.1. Ca^2+^ Signals and mTORC1 Signaling

Changes in intracellular Ca^2+^ concentration have been suggested to affect mTORC1 signaling. Graves et al. reported that the phosphorylation of S6K1, a well-known substrate of mTORC1, was increased by treatment with the Ca^2+^ ionophore A23187 or the sarcoplasmic/endoplasmic reticulum Ca^2+^-ATPase (SERCA) inhibitor thapsigargin [31]. Both treatments resulted in an increase in intracellular Ca^2+^ levels, while the increase in S6K1 phosphorylation was inhibited by the intracellular and extracellular Ca^2+^ chelators, 1,2-bis(2-aminophenoxy)ethane-N,N,N′,N′-tetraacetic acid tetrakis(acetoxymethyl ester) (BAPTA-AM) and ethylene glycol-bis(β-aminoethyl ether)-N,N,N′,N′-tetraacetic acid (EGTA), respectively [31]. Similarly, Conus et al. revealed that treatment with thapsigargin or another Ca^2+^ ionophore, ionomycin, induced the phosphorylation of S6K1. Pretreatment with EGTA completely abolished S6K1 activity under mitogenic stimuli [32]. These studies also showed that these treatments did not have a significant impact on the phosphorylation status of Akt. Similarly, Hannan et al. reported that Ca^2+^ is required for the priming step of S6K1 activation [39]. These initial findings suggest the involvement of Ca^2+^ mobilization in the mTORC1 signaling pathway.

Amino acids are fundamental activators of the mTORC1 pathway (Figure 1). Interestingly, several reports have described a relationship between amino acids and intracellular Ca^2+^ mobilization [19,33,40,41,42,43]. Mercan et al. have reported that leucine administration caused an intracellular Ca^2+^ rise by inducing a release from the endoplasmic reticulum (ER) Ca^2+^ store in an inositol-1,4,5-trisphosphate (IP_3_) receptor (IP_3_R)-dependent manner in C2C12 cells [40]. The intracellular Ca^2+^ rise is likely mediated by the SH2 domain-containing protein tyrosine phosphatase (SHP-2)–phospholipase Cβ4 (PLCβ4) axis [40]. Although the detailed mechanism underlying the involvement of SHP-2 in leucine-induced Ca^2+^ mobilization is unclear, tyrosine phosphatase activity of SHP-2 is essential for mTORC1 activation [40]. In addition, Wauson et al. also reported that the G protein-coupled receptor (GPCR) umami taste receptor taste receptor type 1 member 1 (T1R1)/T1R3 is important in amino acid-induced mTORC1 activation via Ca^2+^ mobilization [41]. Amino acids sensed by T1R1/T1R3 activate mTORC1 through the PLCβ–Ca^2+^ extracellular signal-regulated kinase (ERK)1/2 pathway, resulting in the promotion of protein synthesis and the suppression of autophagy [42]. Moreover, Ca^2+^ influx by activated T1R1/T1R3 is dependent on voltage-dependent L-type Ca^2+^ channels (VDCCs), because the blockade of VDCCs by nifedipine partially disturbs amino acid-induced mTORC1 activation [41].

Interestingly, independent of known mTORC1-activating conditions, such as amino acid availability, an intracellular Ca^2+^ rise can activate mTORC1 [35,44,45]. Receptors for neuropeptides orexin-A and orexin-B (ORX1 and ORX2) appear to mediate the Ca^2+^ influx for mTORC1 activation [44]. The orexin-A and orexin-B neuropeptides are necessary for maintaining arousal. Depletion of these peptides causes the chronic sleep disorder of narcolepsy [46,47]. Wang et al. revealed that mTORC1 activation following orexin sensing by ORX1 and ORX2 is independent of Akt or ERK, but depends on signal transduction through extracellular Ca^2+^ influx and the lysosomal pathway via v-ATPase and Rag GTPase [44]. Furthermore, orexin-dependent Ca^2+^ influx likely occurs through VDCCs, similar to the T1R1/T1R3-mediated influx. Thus, GPCRs for several signaling molecules participate in mTORC1 regulation via Ca^2+^ influx through VDCCs.

mTORC1 regulation by Ca^2+^ signaling has recently emerged from a physiological perspective. In an aging society, sarcopenia and muscle weakness with age are serious problems. In addition to cardiac hypertrophy associated with Ca^2+^-mediated mTORC1 regulation [37,38], Ito et al. revealed that the mechanical load can induce skeletal muscle hypertrophy via Ca^2+^-mediated mTORC1 activation [48]. The mechanical load can induce neuronal nitric oxide synthase (nNOS) activation in skeletal muscle. At the sarcolemma in skeletal muscle, nNOS is associated with the dystrophin complex that links the extracellular matrix to the intracellular cytoskeleton [49]. The mechanical load might activate stretch-activated ion channels, which in turn rapidly activate nNOS by binding with Ca^2+^ and calmodulin (CaM) [49]. Subsequently, nitric oxide (NO) is produced from L-arginine by activated nNOS and is then converted to peroxynitrite, which in turn can cause a rise in intracellular Ca^2+^ through transient receptor potential cation channel, subfamily V, member 1 (TRPV1) located at the sarcoplasmic reticulum [48]. The TRPV1 agonist capsaicin alone can cause skeletal muscle hypertrophy via an increase in intracellular Ca^2+^ levels and mTORC1 activation, suggesting that the TRPV1-mediated intracellular Ca^2+^ rise is important for mTORC1 activation [48,50]. Interestingly, the entry of bacteria-derived Ca^2+^ into host macrophages also appears to upregulate mTORC1 signaling [51]. The P2A-type ATPase CtpF in *Mycobacterium tuberculosis* is a plasma membrane Ca^2+^ transporter essential for survival of the bacteria in host macrophages. In *M. tuberculosis*-infected macrophages, Ca^2+^ efflux through CtpF of *M. tuberculosis* into host macrophages facilitates mTORC1 activation and impairs autophagy, which is thought to be a key mechanism for *M. tuberculosis* to escape elimination in infected cells during the early stages of infection, although the mechanism of how CtpF causes an intracellular Ca^2+^ rise in host macrophages remains unknown [51]. Thus, many reports have linked Ca^2+^ signaling to mTORC1 regulation.

It is also reported that mTOR affects Ca^2+^ signaling as well. For instance, mTOR signaling is involved in diverse biological pathways by regulating the activity and expression of various Ca^2+^ channels [52,53,54]. Platelet-derived growth factor (PDGF) elevates mTOR signaling, which causes an upregulation of stromal interaction molecule 1 (STIM1) and Orai1, an ER Ca^2+^ sensor and a Ca^2+^ channel at the plasma membrane, respectively [52]. These proteins contribute to the store-operated Ca^2+^ entry (SOCE), a Ca^2+^ entry from the extracellular space induced by the depletion of ER Ca^2+^ levels [55]. The subsequent intracellular Ca^2+^ increase through SOCE induces cell proliferation [52]. mTOR has also been reported to interact with lysosomal two-pore segment channel 2 (TPC2) [53,56], and inhibition of mTORC1 by the mTORC1 inhibitor rapamycin induces mobilization of Ca^2+^ from lysosomes through TPC2 [57]. Hyperactivation of mTORC1 might be involved in epilepsy in tuberous sclerosis complex (TSC) patients through the elevation of Ca^2+^ signaling [54]. TSC2 is a potent negative regulator of mTORC1, and depletion of TSC2 causes hyperactivation of mTORC1 (more detailed mechanisms are described in Section 1.2.3). Hisatsune et al. revealed that TSC2-deficient neurons show increased neural activity associated with highly synchronized Ca^2+^ spikes [54]. Interestingly, the expression of Cav1.3, a subtype of VDCCs, is upregulated in TSC2-deficient cells, which is inhibited by rapamycin treatment. The enhancement of the Ca^2+^ influx through Cav1.3 leads to abnormal neurite extension and sustained activation of cAMP response element-binding protein (CREB), which plays an important role in synaptic plasticity [54]. Thus, these studies suggest an important role for mTORC1 in Ca^2+^ signaling and a complicated and tightly intertwined relationship between mTOR signaling and Ca^2+^ signaling.

In the following sections, we will discuss the roles of Ca^2+^-binding proteins as key molecules linking intracellular Ca^2+^ mobilization with mTORC1 regulation.

### 1.2. Calmodulin (CaM) and mTORC1 Signaling

CaM is a ubiquitously expressed EF-hand Ca^2+^ sensor protein. An identical CaM protein is encoded by three genes (*CALM1*, *CALM2*, and *CALM3*) in mammals. The binding of Ca^2+^ to CaM induces a significant conformational change from a closed to an open state, exposing a hydrophobic surface that facilitates the binding with its target protein [58,59]. CaM plays vital roles in a wide range of biological processes, including cell growth [60], cell cycle progression, proliferation [61,62], and trafficking [63] by binding to hundreds of target proteins in a Ca^2+^-dependent manner. For example, CaM regulates smooth muscle contraction by binding and activating myosin light-chain kinase (MLCK) [64]. CaM has also been reported to mediate the activation of the mTOR pathway in response to intracellular Ca^2+^ increases induced by various stimuli. Interestingly, it has been reported that the addition of amino acids enhanced mTORC1 activity through an increase in intracellular Ca^2+^, which is sensed by CaM [19,33,34]. Gulati et al. reported that increased Ca^2+^ levels enhance the interaction between CaM and human vacuolar protein sorting 34 (hVps34), leading to mTORC1 activation [34]. Ca^2+^/CaM also binds to TSC2 and likely inhibits repression of TSC2 toward Rheb, leading to mTORC1 activation [33]. Irrespective of amino acid availability, Ca^2+^/CaM is also reported to directly bind to mTOR upon lysosomal Ca^2+^ release through the lysosome-resident Ca^2+^ channel, the transient receptor potential mucolipin 1 (TRPML1) [35]. In addition, cadmium is a toxic environmental contaminant that can promote Ca^2+^ entry and activation of the mTOR and MAPK pathways, leading to apoptosis [45]. Silencing CaM or pretreatment with BAPTA-AM, EGTA, or a CaM inhibitor, trifluoperazine, can prevent cadmium-induced activation of mTOR signaling, suggesting that Ca^2+^/CaM acts in cadmium-induced activation of mTOR signaling. Thus, it is plausible that Ca^2+^/CaM can modulate mTORC1 activity in concert with other pathways. mTORC1 might act as a coincidence detector, which ensures cell growth and proliferation only when both nutrient levels and cellular status (Ca^2+^ levels) are sufficient.

Some well-known downstream effectors of CaM, CaMKs, also appear to be involved in the regulation of mTORC1 signaling. In fetal mouse neurons, a short-term glutamatergic stimulation reportedly activated the phosphatidylinositol-3 kinase (PI3K)–mTORC1 pathway. The activation requires increased intracellular Ca^2+^ via postsynaptic VDCCs, CaM, and Ca^2+^/CaM-dependent kinase (CaMK) II [65]. The CaM inhibitor calmidazolium and the CaMKII inhibitor KN-93 prevented the increase in S6K1 phosphorylation. In human autosomal dominant polycystic kidney disease cells, the knockdown of CaMK4 or the inhibition of CaMK4 by KN-93 reportedly decreased S6K1 phosphorylation and cell proliferation. Moreover, the inhibition of CaM and CaMK kinase (CaMKK)β, two key upstream regulators of CaMK4, also attenuates mTORC1 signaling [66]. In addition, Song et al. reported that CaMKII can phosphorylate and inactivate glycogen synthase kinase-3 (GSK-3) in neuronal cells [67]. Because the inhibition of GSK3 leads to mTORC1 activation via the repression of TSC2 [68], it is possible that CaMKII might contribute mTORC1 activation through the phosphorylation of GSK3.

As described above, the binding of CaM to target proteins influences the regulation of the mTORC1 pathway in response to changes in Ca^2+^ levels. Because these factors are of interest as central mediators for regulating mTORC1 signaling in response to changes in intracellular Ca^2+^ levels, we discuss these factors in more detail below.

#### 1.2.1. hVps34

**Figure 2:**
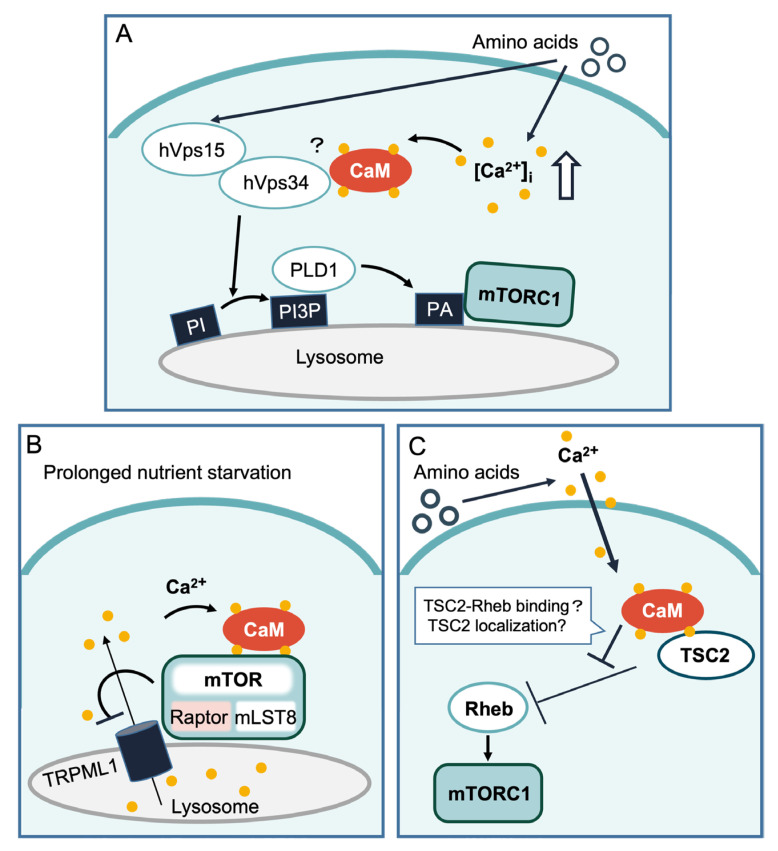
Ca^2+^/CaM-mediated regulation of mTORC1. (**A**) Amino acids have been reported to elevate intracellular Ca^2+^ concentration and enhance hVps34 kinase activity by promoting the binding between hVps34 and CaM in a Ca^2+^-dependent manner. hVps34 has also been suggested to be activated by amino acids by binding to hVps15. Activated hVps34 generates PI3P from PI in the lysosome, which can trigger PLD1 recruitment. PLD1 then produces PA, which leads to mTORC1 activation. (**B**) The lysosomal Ca^2+^ channel TRPML1 is negatively regulated by mTORC1. Prolonged nutrient starvation may decrease mTORC1 activity and relieve its inhibition. TRPML1-mediated Ca^2+^ release from the lysosome is sensed by CaM and might activate mTORC1 by the direct binding of Ca^2+^/CaM to mTOR. (**C**) Amino acids provoke extracellular Ca^2+^ influx. Ca^2+^/CaM activates mTORC1 through the TSC2–Rheb axis by binding to and inhibiting TSC2. Binding of Ca^2+^/CaM to TSC2 might affect TSC2 actions by affecting TSC2 binding to Rheb and/or TSC2 localization, although the detailed mechanism of how Ca^2+^/CaM suppresses TSC2–Rheb remains unclear. Ca^2+^ is indicated by yellow circles.

Gulati et al. originally demonstrated that amino acids, especially leucine, induce a rise in intracellular Ca^2+^, which is required for mTORC1 activity [34]. Mechanistically, the increase in Ca^2+^ levels upon amino acid addition promotes binding between CaM and hVps34 [34], a class III PI3K that generates phosphatidylinositol 3-phosphate (PI3P), leading to the activation of mTORC1 [69,70] (Figure 2A). Several other studies demonstrated that hVps34 participates in the amino acid-induced activation of mTORC1 [71,72]. The addition of amino acids or glucose reportedly increased PI3P levels [70]. The increase in PI3P is suggested to promote the translocation of phospholipase D1 (PLD1) to the lysosome, where the production of phosphatidic acid (PA) facilitates mTORC1 activity [71,72] (Figure 2A). However, whether Ca^2+^/CaM binding to hVps34 is a key mechanism to activate mTORC1 remains controversial [35,73], because hVps15 was reported to be a more plausible candidate to activate hVps34 [73]. In particular, Yan et al. showed that hVps34 activity is regulated by its interaction with the Vps15 subunit of Vps34 kinase, but not by Ca^2+^/CaM, because BAPTA-AM or the CaM inhibitor W-7 did not affect hVps34 activity [73]. Therefore, it remains an open question whether Ca^2+^/CaM primarily contributes to the regulation of mTORC1 activity by directly altering hVps34 activity.

#### 1.2.2. TRPML1 and mTOR

Lysosomes are organelles involved in intracellular Ca^2+^ storage [74]. Mutations in TRPML1 are known to cause a severe lysosomal storage disorder called mucolipidosis type IV [75]. TRPML1 activity is at least in part regulated by membrane lipids. Phosphatidylinositol (3,5)-bisphosphate [PI(3,5)P_2_], a low-abundance phosphoinositide present in late endosomes and lysosomes, can activate TRPML1 [76,77,78]. However, the detailed activation mechanism remains unclear, because PI(3,5)P_2_-bound TRPML1 appears to be in the closed conformation from the structural analysis [79]. TRPML1 modulates mTORC1 activity under nutrient-limited environments [35,36] (Figure 2B). The knockdown of TRPML1 results in the reduction of lysosomal Ca^2+^ efflux and reduced mTORC1 activity. In contrast, the overexpression of TRPML1 or stimulation with the TRPML1 agonist, ML-SA1, elevates mTORC1 activity and attenuates autophagy by inducing an interaction between CaM and mTORC1 in a Ca^2+^-dependent manner [35]. Interestingly, Ca^2+^/CaM binding to mTORC1, most likely to mTOR, was demonstrated to increase mTORC1 kinase activity in vitro [35], although the mTOR binding sites of Ca^2+^/CaM have not yet been identified. Conversely, mTORC1 phosphorylates TRPML1 at Ser572 and Ser576, thereby decreasing its Ca^2+^ efflux activity [80] (Figure 2B). Thus, it seems that mTORC1 and TRPML1 regulate each other’s activity, and this relationship would be helpful for regulating autophagy induction depending on the degree of nutrient starvation. Indeed, prolonged nutrient starvation leads to the reactivation of mTORC1 through CaM and TRPML1 [36]. This reactivation of mTORC1 by TRPML1 may provide minimal mTORC1 activity, even during starvation, to maintain cellular homeostasis [36]. It is also important to note that Ca^2+^ efflux through TRPML1 activity is also associated with the regulation of autophagy, independent of mTORC1 [81,82]. For instance, Ca^2+^/CaM-dependent phosphatase calcineurin is activated by Ca^2+^ released from lysosomes through TRPML1 during starvation, resulting in the dephosphorylation of its target, TFEB. Dephosphorylated TFEB is relieved from sequestration to the cytoplasm, translocates to the nucleus, and promotes the transcription of lysosomal protein genes, including autophagy-related genes [81]. In addition, Ca^2+^ release mediated by TRPML1 also induces autophagic vesicle biogenesis by activating ULK1 and hVps34 through CaMKKβ-AMP-activated protein kinase (AMPK) during the acute phase of autophagy [82]. Therefore, TRPML1-mediated Ca^2+^ release from lysosomes appears to be important for autophagy generally, and the fluctuation of TRPML1 activity resulting from the duration and quality of nutrient starvation is a critical determinant for cells to induce autophagy [83], although it remains largely unclear how autophagy induction by TRPML1-mediated Ca^2+^ release is coordinately regulated. It is already reported that mTORC1 activity is decreased upon the acute phase of nutrient starvation, but its activity becomes reactivated during longer starvation due to a resupply of amino acids by autophagy [84]. Thus, it is possible that the changes of TRPML1 activity in the early phase of starvation might promote autophagy, independent of mTORC1 inactivation, and subsequent reactivation of mTORC1 in the later phase of starvation might then limit TRPML1 activity to readjust cellular status.

#### 1.2.3. Tuberous Sclerosis Complex 2 (TSC2)

The *TSC* gene products TSC2 and TSC1 are tumor suppressors that form a complex with TBC1 domain family member 7 (TBC1D7) [85]. TSC2 possesses GTPase-activating protein (GAP) activity toward Rheb, an activator (in its GTP-bound form) of mTORC1. Therefore, the TSC complex functions as a negative regulator of mTORC1 (Figure 1 and Figure 2C). The TSC–Rheb axis was originally thought to be regulated by growth factors, such as insulin. Insulin activates Akt via PI3K. Activated Akt phosphorylates and inactivates TSC2, leading to mTORC1 activation [86,87]. Several recent studies, including ours, have demonstrated that the TSC2–Rheb axis is also regulated by amino acid availability [27,33,88,89,90,91,92,93,94]. In TSC2 knockout cells, mTORC1 activity is less sensitive to amino acid starvation [33,90]. Moreover, changes in the GTP-loaded Rheb levels in response to amino acid availability have been reported [27,89,91]. Recently, we found that TSC2 regulation by amino acids is mediated by Ca^2+^/CaM [33]. We demonstrated that the intracellular Ca^2+^ rise induced by amino acid addition is sensed by CaM, and that Ca^2+^/CaM interacts with TSC2, leading to mTORC1 activation (Figure 2C). The CaM binding site of TSC2 [33,95] is located within the GAP domain and corresponds to one of the α-helices that supports the TSC2–Rheb interaction [96]. Therefore, Ca^2+^/CaM binding to TSC2 may cause a conformational change in the TSC2 GAP domain, thereby inhibiting the interaction between TSC2 and Rheb. In addition, several reports have suggested a role for Ca^2+^/CaM binding in controlling the cellular localization of TSC2. This region contributes to TSC2 translocation to the nucleus [95,97]. A recent study further suggested that Ca^2+^/CaM induces TSC2 translocation from the membrane to the nucleus, where TSC2 forms a complex with vitamin D receptor (VDR) that partly contributes to the transcription of the VDR target gene, *CYP24A1* [98]. Thus, the change in TSC2 localization and/or binding ability to Rheb induced by Ca^2+^/CaM might be a potential mechanism regulating mTORC1 activity.

Regarding the connection between TSC2 and pathology, an autosomal dominant disorder of TSC affects multiple organs, including the brain, skin, kidneys, heart, and lungs, due to inactivating mutations in the *TSC1* or *TSC2* gene. Because TSC2 acts as a negative regulator of mTORC1, mTORC1 inhibitors can combat TSC pathologies, such as hamartomas, caused by mTORC1 hyperactivation [99,100,101]. Furthermore, hyperactivation of mTORC1 in TSC2 knockout mice induces cardiac hypertrophy [102]. Consistently, prolonged aberrant intracellular Ca^2+^ increases cause cardiac hypertrophy by evoking mTORC1 activation [37]. These findings implicate the control of binding between Ca^2+^/CaM and TSC2 as a potential therapeutic target for these diseases. Mutations in *TSC* genes also cause lymphangioleiomyomatosis (LAM) and consequent lung disease, which often occur in women of childbearing age. Because estrogen and progesterone receptors are often expressed in LAM cells, these receptors are considered to be important mediators associated with the cause of LAM [103,104,105]. CaM binding to the estrogen receptor has been reported to enhance the stability of the estrogen receptor [106], although the detailed mechanism is unclear. Interestingly, the Ca^2+^/CaM-binding region of TSC2 overlaps with the estrogen receptor-binding region of TSC2 [107]. It is possible that Ca^2+^/CaM binding to TSC2 modulates the estrogen receptor stability, and deletion of TSC2 might trigger the onset of LAM by simultaneously affecting the estrogen receptor stability. Thus, the understanding of the mutual interactions of CaM, TSC2, and the estrogen receptor would provide important insight into the pathology of this disease.

## 2. Ca^2+^/CaM and mTORC2 Signaling

**Figure 3:**
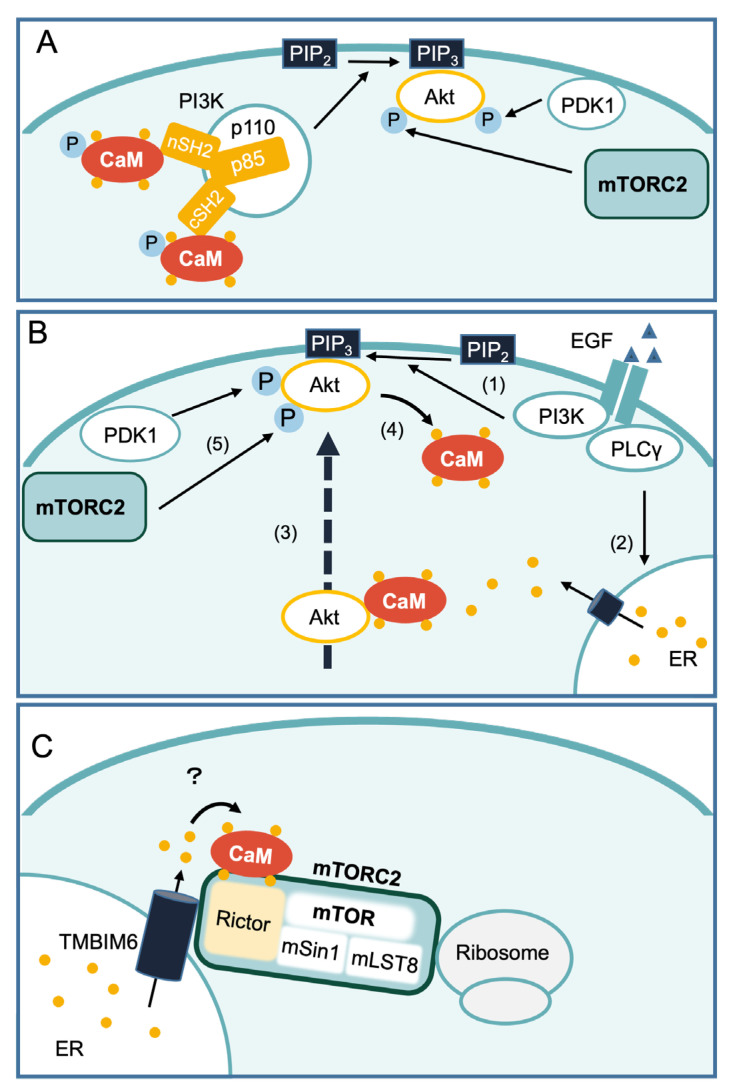
Potential mechanisms of Ca^2+^/CaM-mediated regulation of Akt and mTORC2. (**A**) Ca^2+^/CaM phosphorylated at Tyr99 interacts with the N-terminal domain (nSH2) and the C-terminal domain (cSH2) of the 85kDa regulatory subunits of PI3K (p85) and stimulates kinase activity of PI3K. The generated PIP_3_ recruits Akt and PDK1 to the plasma membrane. Akt is fully activated by two kinases, PDK1 and mTORC2. (**B**) EGF induces an intracellular Ca^2+^ rise and enhances Akt activity by promoting CaM–Akt binding as follows: (1) EGF binding to EGFR activates PI3K to generate PIP_3_ from PIP_2_. (2) EGFR also activates PLCγ and induces Ca^2+^ release from the ER via an IP_3_-dependent mechanism. (3) Binding of Ca^2+^/CaM to the PH domain of Akt is proposed to change the conformation of Akt and to facilitate its targeting to the plasma membrane. (4) Binding of Akt to PIP_3_ promotes the dissociation from CaM. (5) Akt is phosphorylated by PDK1 and mTORC2 and is fully activated. (**C**) TMBIM6 Ca^2+^ channel-like protein interacts with mTORC2. Ca^2+^ released from the ER through TMBIM6 is necessary for mTORC2 assembly and association with ribosomes. Because CaM binds to Rictor in a Ca^2+^-dependent manner [33], CaM may mediate Ca^2+^ signaling to mTORC2 activation. Ca^2+^ is indicated by yellow circles.

Compared with the regulation of the mTORC1 pathway by Ca^2+^/CaM, few studies have examined the regulation of the mTORC2 pathway by Ca^2+^/CaM. However, PI3K and Akt, the upstream and downstream factors of mTORC2, respectively, are reportedly regulated by Ca^2+^/CaM (Figure 3A,B). In 1997, Ca^2+^/CaM was shown to enhance PI3K activation through its interaction with the Src homology 2 (SH2) domain in the 85 kDa regulatory subunit of PI3K [108]. Subsequent analyses revealed the detailed mechanisms. CaM phosphorylated at Tyr99 first binds to the C-terminal domain of the p85 subunit (cSH2), and then to the N-terminal domain (nSH2) with a time lag [109,110] (Figure 3A). The insulin receptor tyrosine kinase phosphorylates CaM at Tyr99 in vitro. This phosphorylation generally increases the interaction between CaM and its target proteins [111]. Ca^2+^/CaM also directly contributes to Akt regulation (Figure 3B). Deb et al. revealed that Akt activation by the epidermal growth factor (EGF) was attenuated upon treatment with PLC inhibitor U-73122, BAPTA-AM, or CaM inhibitor W-7 in c-Myc-overexpressing mammary carcinoma cells [112]. Because treatment with W-7 did not decrease PI3K activation and treatment with CaMK, CaMKII, or CaMKIII inhibitors did not affect EGF-stimulated Akt activation, the involvement of Ca^2+^/CaM in Akt activation was thought to be a direct, rather than indirect, regulation via PI3K or CaMK. Indeed, Ca^2+^/CaM can bind to Akt at the first 42 amino acid residues within the pleckstrin homology (PH) domain [113]. Furthermore, the addition of EGF reportedly recruited both CaM and Akt to the plasma membrane, which was suppressed by W-7 treatment, in breast cancer cell lines [114]. It is proposed that the binding of CaM with the PH domain of Akt disrupts its interaction with the kinase domain, promoting the translocation of Akt to the plasma membrane [115]. At the plasma membrane, the PH domain of Akt binds to phosphatidylinositol (3,4,5)-trisphosphate (PIP_3_), a product of PI3K activation from phosphatidylinositol (4,5)-bisphosphate [PI(4,5)P_2_]. The binding of Akt with PIP_3_ allows phosphorylation and activation by mTORC2 and 3-phosphoinositide-dependent protein kinase 1 (PDK1). Because CaM was also reported to compete with PIP_3_ for binding to the PH domain of Akt [113,116], the presence of PIP_3_ at the plasma membrane likely promotes dissociation of CaM from Akt [115] (Figure 3B). In addition to the direct effect of CaM on Akt, it remains unclear whether the Ca^2+^/CaM-dependent upregulation of Akt phosphorylation occurs through changes in mTORC2 activity.

Interestingly, recent reports have suggested that changes in Ca^2+^ levels affect mTORC2 activation. It has been suggested that mTORC2 is regulated by nutrients, such as amino acids, glucose, and stress, in addition to growth factors [117,118,119,120]. Ammonium ion is a metabolic waste product that may connect the change in Ca^2+^ levels to mTORC2 regulation. Glutaminolysis contributes to tumor growth, offering fuel for rapid cancer growth while emitting a large amount of ammonium ion. Merhi et al. discovered that ammonium ion could trigger rapid Rictor-dependent mTORC2 activation [121]. Consistent with the report that ammonium ion treatment increased intracellular Ca^2+^ levels transiently [122], BAPTA-AM treatment decreased ammonium ion-stimulated phosphorylation of Akt at Ser473 and phosphorylation of NDRG1 at Thr346, a downstream effector of SGK1, suggesting that ammonium ion-induced mTORC2 activation is dependent on the change of intracellular Ca^2+^ levels. In addition, a recent study provided direct evidence that mTORC2 assembly and activity are regulated by Ca^2+^ signaling [123]. Transmembrane B cell lymphoma 2-associated X protein (Bax) inhibitor motif-containing 6 (TMBIM6, also known as Bax inhibitor 1) contains six transmembrane regions and a Ca^2+^-channel pore domain in its C-terminal region and functions as a Ca^2+^ leak channel at the ER membrane [124] (Figure 3C). TMBIM6 deletion results in reduced mTORC2 activity and Akt-dependent metabolic processes. In particular, TMBIM6 binds directly to Rictor and ribosomes [123] that are required for mTORC2 activation [125]. Moreover, mTORC2 assembly and mTORC2 association with ribosomes can be disrupted in TMBIM6^D213A^ mutant cells in which Ca^2+^ leakage from the ER does not occur [123]. Thus, Ca^2+^ leakage into the cytosol via TMBIM6 appears to be necessary for mTORC2 activity (Figure 3C). However, how exactly Ca^2+^ is sensed by mTORC2 remains unclear. We previously showed that CaM can bind to Rictor as well as TSC2 in a Ca^2+^-dependent manner [33] (Figure 3C). These findings suggest that CaM might bind to mTORC2 directly and mediate Ca^2+^ signals to mTORC2.

## 3. Involvement of Other Ca^2+^ Sensor Proteins in the mTOR Pathway

S100 proteins, named after the solubility of approximately 10,000 Da proteins in 100% saturated ammonium sulfate, are the Ca^2+^-binding protein superfamily that is involved in various biological processes by functioning in both intracellular and extracellular spaces, although most Ca^2+^ sensor proteins, such as CaM, act exclusively in the intracellular space [126,127]. S100 proteins have 25 known family members. The abnormal expression of some of them is frequently observed as a feature of various cancers [126,128]. Recent studies have demonstrated that the mTOR pathway participates in the progression of cancer and other diseases associated with S100 protein dysregulation [129,130,131,132]. For instance, S100A11 knockdown causes a decrease in phosphorylated PI3K, phosphorylated Akt, and phosphorylated mTOR and disturbs cell proliferation and migration in head and neck carcinoma cells, indicating that the upregulation of S100A11 activates the PI3K–Akt-mTOR pathway in these tumor cells [129]. S100A11 was also reported to be a positive regulator of mTOR signaling, contributing to nonalcoholic fatty liver disease [130]. Similarly, S100B increases proliferation and invasion of human colon adenocarcinoma cells, and S100A8/9 activates proliferation and angiogenesis in human umbilical vein endothelial cells through the mTOR pathway [131,132]. However, the detailed molecular mechanism and involvement of changes in Ca^2+^ levels remain largely unexplored.

Like CaM or S100 proteins, neuronal calcium sensor 1 (NCS1) belongs to the EF-hand superfamily of Ca^2+^-binding proteins. NCS1 was initially thought to be expressed only in neuronal cells. However, subsequent evidence revealed its expression in almost all tissues. Abnormally high NCS1 levels are observed in some tumor tissues, contributing to cancer cell survival and migration [133,134]. Recently, Grosshans et al. revealed that oxidative stress or tumor necrosis factor-α activates the transcription factor nuclear factor-kappa B (NF-κB), leading to the upregulation of the transcription level of NCS1 [135]. NCS1 maintains low intracellular Ca^2+^ levels under basal conditions. However, NCS1 increases Ca^2+^ release from the ER in an IP_3_-dependent manner in the presence of environmental stress stimuli, enhancing Akt activity to promote cell survival and motility [135].

Calpains are the family of neutral cysteine proteases that possess the Ca^2+^-binding domain and are directly activated by Ca^2+^. Fifteen isoforms of calpain have been identified in mammals [136]. Conventional calpains (calpain-1 and calpain-2) are ubiquitously expressed and function as heterodimers composed of a catalytic subunit (CAPN1 or CAPN2) and a regulatory subunit (CAPNS1, also known as CAPN4). Caplain-1 or calpain-2 is reported to affect mTOR signaling in physiological and pathological responses [137,138]. Asthma is a chronic airway disease, characterized by several features including airway remodeling. Rao et al. demonstrated that calpains mediate airway muscle cell remodeling through cytokine-induced collagen-I synthesis and proliferation through activation of Akt in mouse asthma models [137]. Indeed, the remodeling was diminished in *capns1* knockout mice, or upon treatment with a selective calpain inhibitor MDL28170, or by the mTOR inhibitor Torin1. Calpain-2 also contributes to brain-derived neurotrophic factor (BDNF)-induced dendritic protein synthesis, which is suggested to be an important role in memory formation [138]. Calpain-2 likely contributes to the activation of mTORC1 and mTORC2 by promoting TSC1, TSC2, and phosphatase and tensin homolog deleted on chromosome 10 (PTEN) degradation, although the detailed mechanism remains unclear.

These observations indicate that Ca^2+^-binding proteins other than CaM may also regulate cell proliferation and survival through the regulation of mTOR activity. Compared with the knowledge of the CaM-mediated regulation of mTOR signaling, the detailed molecular mechanisms of the non-CaM proteins remain to be clarified. Future studies on mTOR regulatory mechanisms, especially in which Ca^2+^-binding proteins are involved, will expand our understanding of the regulation of mTOR signaling.

## 4. Conclusions

Both mTOR signaling and Ca^2+^ signaling are fundamental pathways to regulate cellular adaptation to a wide array of environmental cues. As discussed above, novel regulatory modes of mTORC1 and mTORC2 in response to global or local changes of intracellular Ca^2+^ have emerged. In addition, several studies have further unveiled that Ca^2+^-mediated regulation of mTOR signaling is physiologically and pathologically relevant. Although our knowledge is still limited on how and when Ca^2+^-mediated mTOR regulation is coordinated to maintain cellular homeostasis, further molecular identification of new regulators in cooperation with Ca^2+^-binding proteins will greatly improve our understanding of the complex signaling network under various conditions. Although CaM antagonists are already available, it seems difficult to use those for pharmacological inhibitors of mTOR signaling due to the lack of specificity. Instead, it will be valuable to find a medicine to specifically inhibit CaM binding to target proteins, such as TSC2. In addition to drug screening, the development of a peptide with the binding motif sequence that selectively disrupts CaM–target interaction will be one of the potential clues to combat diseases associated with aberrant Ca^2+^ levels.

## Data Availability

Not applicable.

## Abbreviations

AGC	cAMP-dependent, cGMP-dependent, and protein kinase C-type
AMPK	AMP-activated protein kinase
Atg13	autophagy-related protein 13
BAPTA-AM	1,2-bis(2-aminophenoxy)ethane-N,N,N’,N’-tetraacetic acid tetrakis(acetoxymethyl ester)
Bax	B cell lymphoma 2-associated X protein
BDNF	brain-derived neurotrophic factor
Ca^2+^	calcium ion
CaM	calmodulin
CaMK	Ca^2+^/CaM-dependent protein kinase
CaMKK	CaMK kinase
CREB	cAMP response element-binding protein
ER	endoplasmic reticulum
EGF	epidermal growth factor
EGTA	ethylene glycol-bis(β-aminoethyl ether)-N,N,N′,N′-tetraacetic acid
eEF2K	eukaryotic elongation factor 2 kinase
eIF4A	eukaryotic initiation factor 4A
eIF4B	eukaryotic initiation factor 4B
eIF4E	eukaryotic initiation factor 4E
4E-BP1	eIF4E-binding protein 1
ERK	extracellular signal-regulated kinase
GAP	GTPase-activating protein
GPCR	G protein-coupled receptor
GSK3	glycogen synthase kinase 3
hVps34	human vacuolar protein sorting 34
IP_3_	inositol-1,4,5-trisphosphate
IP_3_R	inositol-1,4,5-trisphosphate receptor
IP_4_	inositol 1,3,4, 5-tetrakisphosphate
LAM	lymphangioleiomyomatosis
mLST8	mammalian lethal with SEC13 protein 8
mSin1	mammalian stress-activated protein kinase-interacting protein 1
mTOR	mechanistic target of rapamycin
mTORC1	mTOR complex 1
mTORC2	mTOR complex 2
MLCK	myosin light-chain kinase
NCS1	neuronal calcium sensor 1
NF-κB	nuclear factor-kappa B
nNOS	neuronal nitric oxide synthase
PA	phosphatidic acid
PDGF	platelet-derived growth factor
PDK1	3-phosphoinositide-dependent protein kinase 1
PIP_2_	phosphatidylinositol (4,5)-bisphosphate
PI3K	phosphatidylinositol-3 kinase
PI3P	phosphatidylinositol 3-phosphate
PI(3,5)P_2_	phosphatidylinositol (3,5)-bisphosphate
PI(4,5)P_2_	phosphatidylinositol (4,5)-bisphosphate
PIP_3_	phosphatidylinositol (3,4,5)-trisphosphate
PLC	phospholipase C
PLD1	phospholipase D1
PH	pleckstrin homology
PKB	protein kinase B
PKC	protein kinase C
PTEN	phosphatase and tensin homolog deleted on chromosome 10
Rag	Ras-related GTP-binding protein
Raptor	the regulatory associated protein of mTOR
Rheb	Ras homolog enriched in brain
Rictor	the rapamycin-insensitive companion of mTOR
S6K1	p70 S6 kinase 1
SERCA	sarcoplasmic/endoplasmic reticulum Ca^2+^-ATPase
SGK1	serum- and glucocorticoid-induced kinases 1
SLC38A9	solute carrier family 38 member 9
SH2	Src homology 2
SHP-2	SH2 domain-containing protein tyrosine phosphatase
SREBP1	sterol regulatory element-binding protein 1
STIM1	stromal interaction molecule 1
T1R1	taste receptor type1 member1
TBC1D7	TBC1 domain family member 7
TFEB	transcription factor EB
TRPV1	transient receptor potential cation channel, subfamily V, member 1
TRPML1	transient receptor potential mucolipin 1
TMBIM6	Transmembrane B cell lymphoma 2-associated X protein (Bax) inhibitor motif-containing 6
TPC2	two-pore segment channel 2
TSC2	tuberous sclerosis complex 2
ULK1	unc-51-like autophagy-activating kinase 1
v-ATPase	vacuolar-type H^+^-ATPase
VDR	vitamin D receptor
VDCCs	voltage-dependent L-type Ca^2+^ channels
